# Finding of widespread viral and bacterial revolution dsDNA translocation motors distinct from rotation motors by channel chirality and size

**DOI:** 10.1186/2045-3701-4-30

**Published:** 2014-06-01

**Authors:** Gian Marco De-Donatis, Zhengyi Zhao, Shaoying Wang, Lisa P Huang, Chad Schwartz, Oleg V Tsodikov, Hui Zhang, Farzin Haque, Peixuan Guo

**Affiliations:** 1 Nanobiotechnology Center, University of Kentucky, Lexington, KY, USA; 2 Department of Pharmaceutical Sciences, College of Pharmacy, University of Kentucky, Lexington, KY, USA; 3 Markey Cancer Center, University of Kentucky, Lexington, KY, USA; 4 Current address: Institute for Biomarker Research, Medical Diagnostic Laboratories, L.L.C., Hamilton, NJ 08690, USA; 5 William Farish Endowed Chair in Nanobiotechnology, School of Pharmacy, University of Kentucky, 565 Biopharmaceutical Complex, 789 S. Limestone Street, Lexington, KY 40536, USA

**Keywords:** DNA translocase, DsDNA viruses, Viral DNA packaging motor, Viral assembly, Bacteriophage, DNA helicase, Revolution force, Phi29, FtsK, RecA, Nanomotor

## Abstract

**Background:**

Double-stranded DNA translocation is ubiquitous in living systems. Cell mitosis, bacterial binary fission, DNA replication or repair, homologous recombination, Holliday junction resolution, viral genome packaging and cell entry all involve biomotor-driven dsDNA translocation. Previously, biomotors have been primarily classified into linear and rotational motors. We recently discovered a third class of dsDNA translocation motors in Phi29 utilizing revolution mechanism without rotation. Analogically, the Earth rotates around its own axis every 24 hours, but revolves around the Sun every 365 days.

**Results:**

Single-channel DNA translocation conductance assay combined with structure inspections of motor channels on bacteriophages P22, SPP1, HK97, T7, T4, Phi29, and other dsDNA translocation motors such as bacterial FtsK and eukaryotic mimiviruses or vaccinia viruses showed that revolution motor is widespread. The force generation mechanism for revolution motors is elucidated. Revolution motors can be differentiated from rotation motors by their channel size and chirality. Crystal structure inspection revealed that revolution motors commonly exhibit channel diameters larger than 3 nm, while rotation motors that rotate around one of the two separated DNA strands feature a diameter smaller than 2 nm. Phi29 revolution motor translocated double- and tetra-stranded DNA that occupied 32% and 64% of the narrowest channel cross-section, respectively, evidencing that revolution motors exhibit channel diameters significantly wider than the dsDNA. Left-handed oriented channels found in revolution motors drive the right-handed dsDNA via anti-chiral interaction, while right-handed channels observed in rotation motors drive the right-handed dsDNA via parallel threads. Tethering both the motor and the dsDNA distal-end of the revolution motor does not block DNA packaging, indicating that no rotation is required for motors of dsDNA phages, while a small-angle left-handed twist of dsDNA that is aligned with the channel could occur due to the conformational change of the phage motor channels from a left-handed configuration for DNA entry to a right-handed configuration for DNA ejection for host cell infection.

**Conclusions:**

The revolution motor is widespread among biological systems, and can be distinguished from rotation motors by channel size and chirality. The revolution mechanism renders dsDNA void of coiling and torque during translocation of the lengthy helical chromosome, thus resulting in more efficient motor energy conversion.

## Background

Transportation of dsDNA from one cellular compartment to another is a prevalent process in all living systems. Many members of the ASCE (Additional Strand Catalytic E) superfamily are nanomotors with a hexameric arrangement of subunits that facilitate a wide range of functions, including dsDNA riding, tracking, packaging, and translocation, which are critical to many processes such as DNA repair, replication, recombination, chromosome segregation, transcription, and cellular reorganization [1,2]. Despite their functional diversity, a common feature of the motors of this family is their ability to convert energy obtained from the binding or hydrolysis of ATP into mechanical energy which results in local/global protein unfolding, complex assembly/disassembly, or grabbing/pushing dsDNA for translocation [1-11]. The hexagonal shape of the motor facilitates bottom-up assembly in nanomachine manufacturing [12-20].

**Figure 1:**
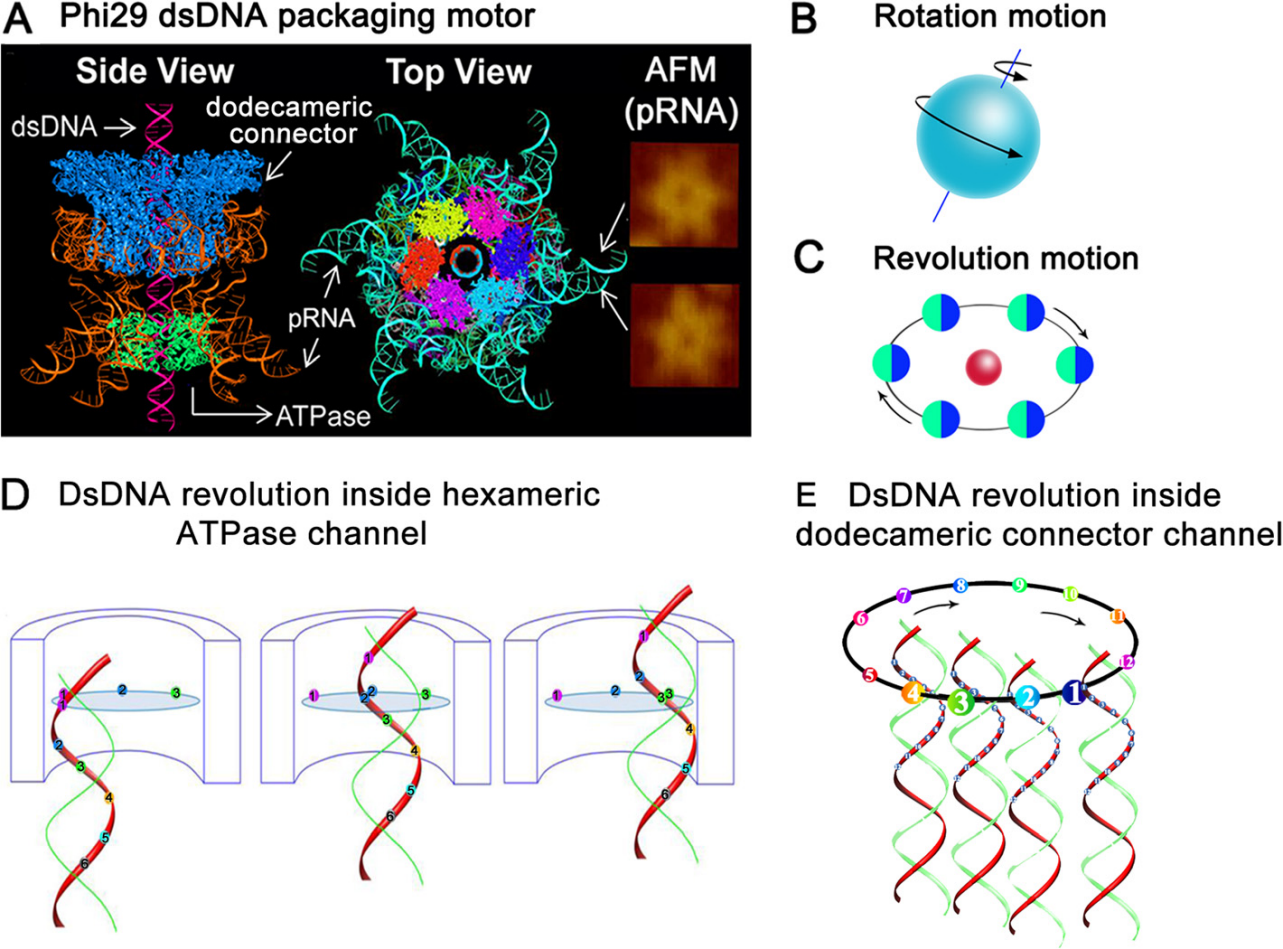
**Illustration of rotation motions and revolution motions using Phi29 revolution motor as an example. (A)** 3D structure of Phi29 dsDNA packaging motor in a side view and top view with a pRNA hexamer derived from the crystal structure [16], and the AFM images of the pRNA hexamer with extended loops. **(B)** Illustration of rotation motors like the Earth *rotates* around its own axis. **(C)** Illustration of revolution motors like the Earth *revolves* around the Sun without rotation. **(D)** Illustration of the dsDNA revolution inside the hexameric ATPase channel. Only three of the six steps are shown. **(E)** Illustration of the dsDNA revolution inside the dodecameric connector channel, only four of the twelve steps are shown. Neither the channel nor the dsDNA needs to rotate during the revolution through channels (for animation, see http://nanobio.uky.edu/Connector-DNA revolution.wmv).

Nanobiomotors have previously been classified into two main categories: linear and rotational motors, which have been clearly documented using single-molecule imaging and X-ray crystallography [21-26]. During replication, dsDNA viruses translocate their genomic DNA into preformed protein shells (procapsids) [27-33]. This entropically unfavorable process is accomplished by a nanomotor that uses ATP as an energy source [34-42]. This dsDNA packaging motor consists of a connector channel and packaging molecules to carry out its activities. For 35 years, it has been popularly believed that DNA packaging in dsDNA viruses involves rotation motors [43], which is seemingly supported by the swivel structure in the crystal structures of all connector channels of bacteriophages [44-46]. However, extensive investigations revealed that the dsDNA packaging motor channels do not rotate during motor actions [47-51]. For example, the T4 DNA-packaging motor remains active when the motor channel protein is crosslinked to the protein shell [47]. Single-molecule imaging further verified that there is no rotation of the channel during packaging [48]. These evidences have brought up a puzzle concerning how packaging can involve a rotation motor without any rotating components. In 2010, another question was raised regarding the inverse orientations of the Phi29 motor channel and dsDNA helices [52], which further questioned the involvement of rotational motion, since the rotation mechanism of dsDNA as a bolt threading onto a motor channel as a nut requires that the threads of the bolt and the nut have the same directionality. Recently, we have discovered that bacteriophage Phi29 dsDNA packaging motor uses a revolution mechanism without rotation, coiling, or torque forces (Figure 1) [9,50,53,54]. The hexameric ATPase ring exercises a force to push the dsDNA through the dodecamer channel acting as a one-way valve [9,10,52]. Observation of this revolution mechanism establishes a third class of biomotors. This finding resolves many puzzles throughout the history of long-lasting studies on the motor [9,10,55].

As the translocation of dsDNA is a ubiquitous process in living systems, and motors of all dsDNA bacteriophages share some common structural and functional features, we aimed at determining whether the revolution model discovered in Phi29 can generally be applied to other DNA packaging motors. Cellular counterparts that show a strong similarity to the Phi29 viral DNA packaging motor are the FtsK and SpoIIIE family motors, featuring a hexameric motor that transports DNA and separates the intertwined lengthy genomic dsDNA during cell division or binary fission [56-63]. Unwinding of the supercoiled dsDNA resulting from rotation would lead to expensive energy consumption [64]. The revolution mechanism adopted by biological systems during evolution, resembles an optimized mechanism for translocation of lengthy dsDNA genome without coiling. In this report, we analyze the motor mechanism regarding force generation of Phi29 and compare its structure and mechanism to that of DNA packaging motors of SPP1, P22, T7, HK97, mimivirus, and vaccinia virus, as well as some cellular proteins such as FtsK and SpoIIIE. We also provide a simple way to distinguish between revolution and rotation motors by channel size and chirality.

## Results and discussion

### Revolution and rotation motors can be distinguished by motor channel size

**Figure 2:**
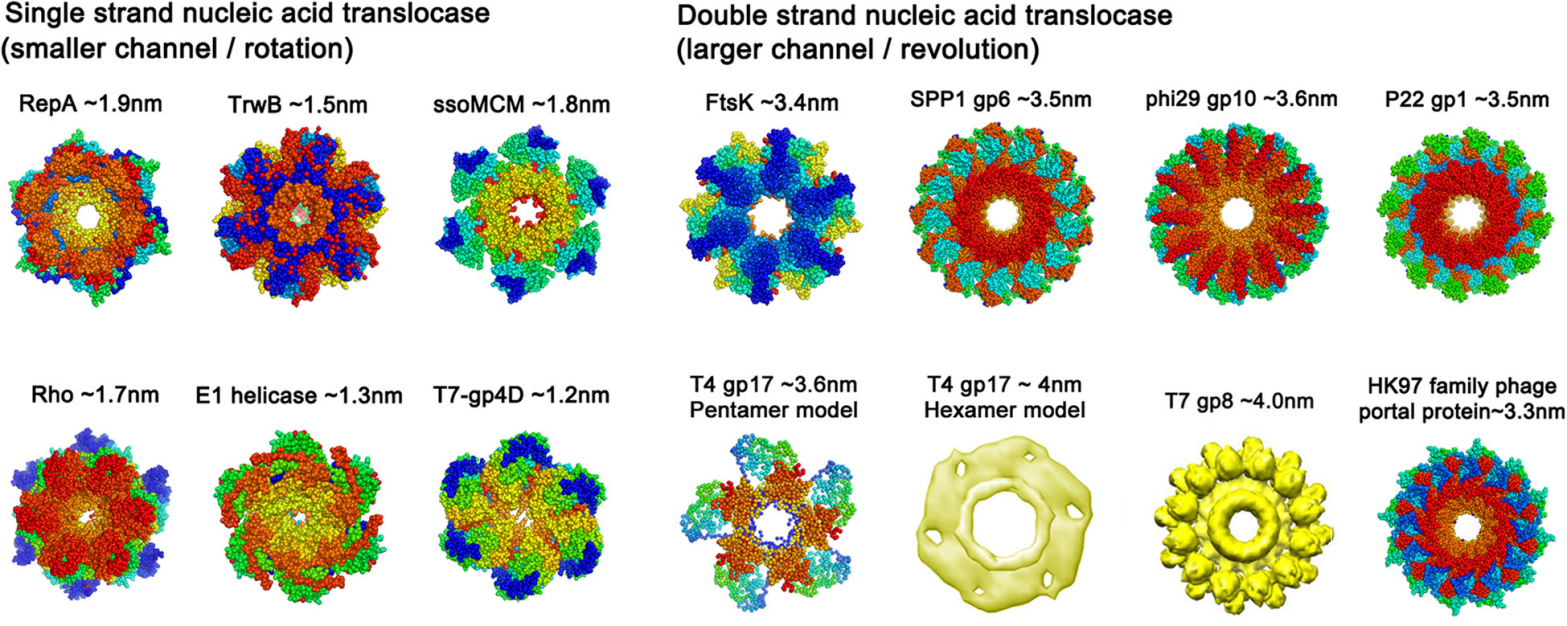
**Comparison of the channel sizes between biomotors using rotation mechanism (left panel) and revolution mechanism (right panel).** The motor channel of dsDNA phages shown in the right panel all have a channel size twice the width of dsDNA, make it impossible for a bolt and nut treading mechanism to work, thus supporting revolution rather than rotation mechanism. (PDB: RepA, 1G8Y; TrwB: 1E9R; ssoMCM, 2VL6; Rho, 3ICE; E1, 2GXA; T7-gp4D, 1E0J; FtsK, 2IUU; Phi29-gp10, 1H5W; HK97 family-portal protein, 3KDR; SPP1-gp6, 2JES; P22-gp1, 3LJ5; T4-gp17, 3EZK). The pentamer and hexamer models of T4 ATPase gp17 display a channel of 3.6 and 4.0 nm, respectively [66].

Previous observations that only one subunit of the hexamer binds to dsDNA at a time [8,50], as well as the cooperativity and sequential action among hexameric ATPase subunits [8], confirmed the revolution of dsDNA along the channel [50]. In this revolution process, dsDNA advances by sliding along the channel wall instead of proceeding through the center of the channel. Thus, the channel would be expected to be wider than the diameter of the dsDNA to ensure sufficient space for revolution. Inspection of the motor channel size in available crystal structures and cryo-EM data confirmed this expectation; while the width of dsDNA is 2 nm, the diameters of the narrowest region of the connector channels of Phi29 [46], SPP1 [65], HK97, the ATPase ring of T4 [66], as well as the dsDNA translocase FtsK [60] of bacteria, are all larger than 3 nm (Figure 2).

On the other hand, the channels of rotation motors, such as replicative DNA helicases TrwB, E1, and DnaB [67-69], are smaller than 2 nm in diameter (Figure 2). For rotation motors, the channel would thus be expected to have a similar width as the ssDNA to allow for the bolt and nut threading mechanism. Nonetheless, during some processes for certain rotation motors, only one strand enters the channel while the other remains outside [5,64,67-71]. In these situations, local unwinding fluctuations of the dsDNA might cause separation of the two strands and facilitate the threading of the ssDNA strand into the center of the hexameric ring, as suggested by smFRET experiments [72-74]. It has been reported that the ssDNA within the channel displays an A form helical structure [69], thus the channel diameter should be no larger than 2 nm to allow for contact between the DNA and the channel. The situation for branch migration is more complicated and beyond the scope of this manuscript. Overall, the above data indicates that the revolution motor can be distinguished from the rotation motor by the size of the motor channel.

### Conductance assay of single connector channels for translocation of tetra-stranded DNA reveals a three-fold width of Phi29 channels compared to dsDNA

**Figure 3:**
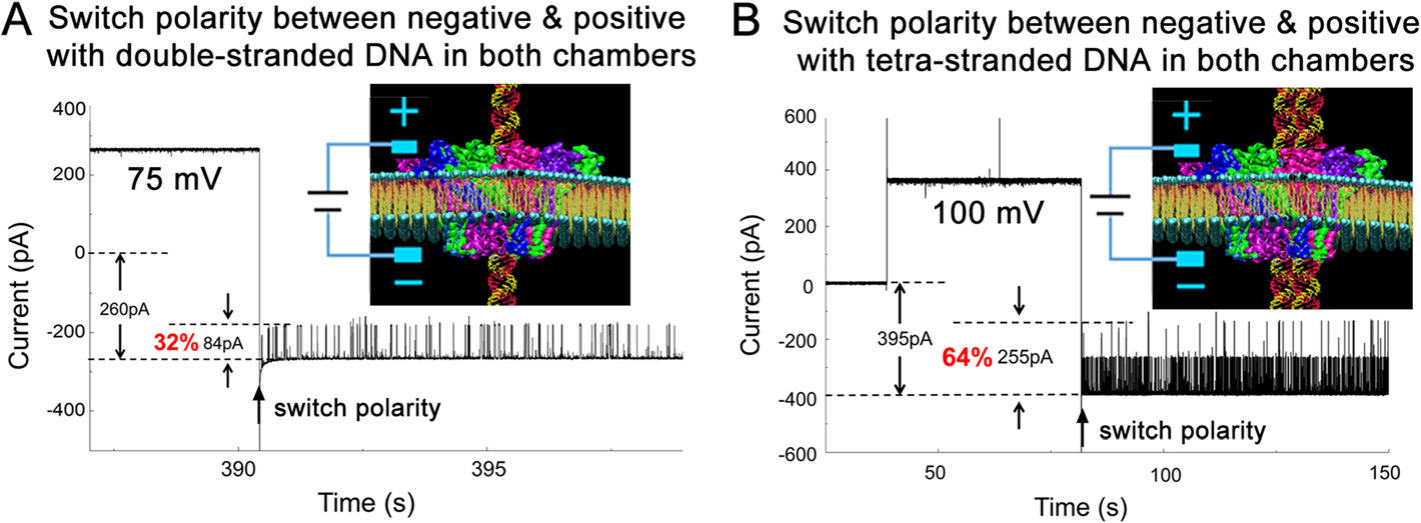
**Demonstration of Phi29 connector channel size larger than the diameter of dsDNA as proved by single pore translocation of double-stranded and tetra-stranded DNA by conductance assay.** Current blockage of the channel by double-stranded was 32% **(A)** and by tetra-stranded DNA was 64% **(B)**. Purified Phi29 connector was incorporated into lipid bilayer. Switch of voltage polarity revealed that the channel allowed only unidirectional translocation of both dsDNA **(A)** and tetra-stranded DNA **(B)**.

The channel size was further assessed by single-channel conductance assays using Phi29 connector channels as a model system. A current blockage of 32% was observed for translocation of dsDNA through the connector channel (Figure 3A), consistent with the ratio of the cross-sectional areas of dsDNA ((2/2)^2^ × 3.14 = 3.14 nm^2^) and channel ((3.6/2)^2^ × 3.14 = 10.2 nm^2^, 10.2 nm^2^ ÷ 3.14 nm^2^ = 32%). For tetra-stranded DNA, which was constructed by DNA nanotechnology (Materials and methods) (Haque F, Wang S, Stites C, Chen L, Wang C, Guo P: Single pore translocation of folded, double-stranded, and tetra-stranded DNA through channel of bacteriophage Phi29 DNA packaging motor, submitted), when passing through the connector channel, a blockage of ~64% was observed (Figure 3B). Thus, the cross-sectional area at the narrowest region of the Phi29 connector funnel is three-fold the area of the dsDNA. Such a big channel size makes it impossible for a bolt and nut tracing mechanism, and makes it likely that only one ATPase subunit at a time can bind to dsDNA [8,50].

Both dsDNA and tetra-stranded DNA show one-way translocation through the Phi29 motor channel, since the switch of the electrical polarity changed the dsDNA from passable to impassable or vice versa through the channel (Figure 3). One-way traffic of tetra-stranded DNA reveals that the channel does not merely serve as a pathway, it plays an active role by forming contacts with translocating double- and tetra-stranded DNA.

### The left-handed chirality of revolution motors is distinct from the right-handed chirality of rotation motors

**Figure 4:**
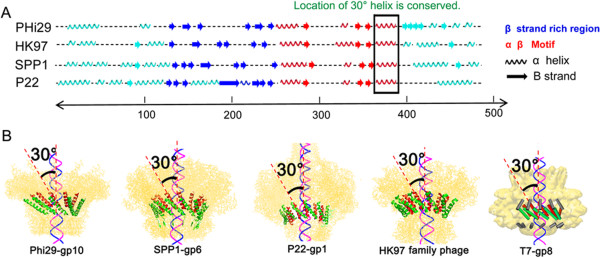
**Secondary and crystal structures of motor channels of different bacteriophages demonstrating left-handed channel wall with a twisting of 30° leftward. (A)** The 3D structure of Phi29 (P04332), HK97 (Q6NFR1), SPP1 (P54309), P22 (P26744) were predicted using the program PredictProtein with default parameter (http://www.predictprotein.org), revealing the 30° left-handed regions correlated well with their respected crystal structures (PDB:Phi29-gp10, 1H5W; HK97 family-portal protein, 3KDR; SPP1-gp6, 2JES; P22-gp1, 3LJ5). The location of the 30° left-handed tilted helix in each bacteriophage connector protein subunit is framed, which all lay at the end of the α β motif. **(B)** The 30° tilt helix (red) is also shown in an external view in connector 3D structures of different bacteriophages, supporting the common mechanism that DNA revolves through the 30° tilted channel by an anti-chiral arrangement in dsDNA translocation.

**Figure 5:**
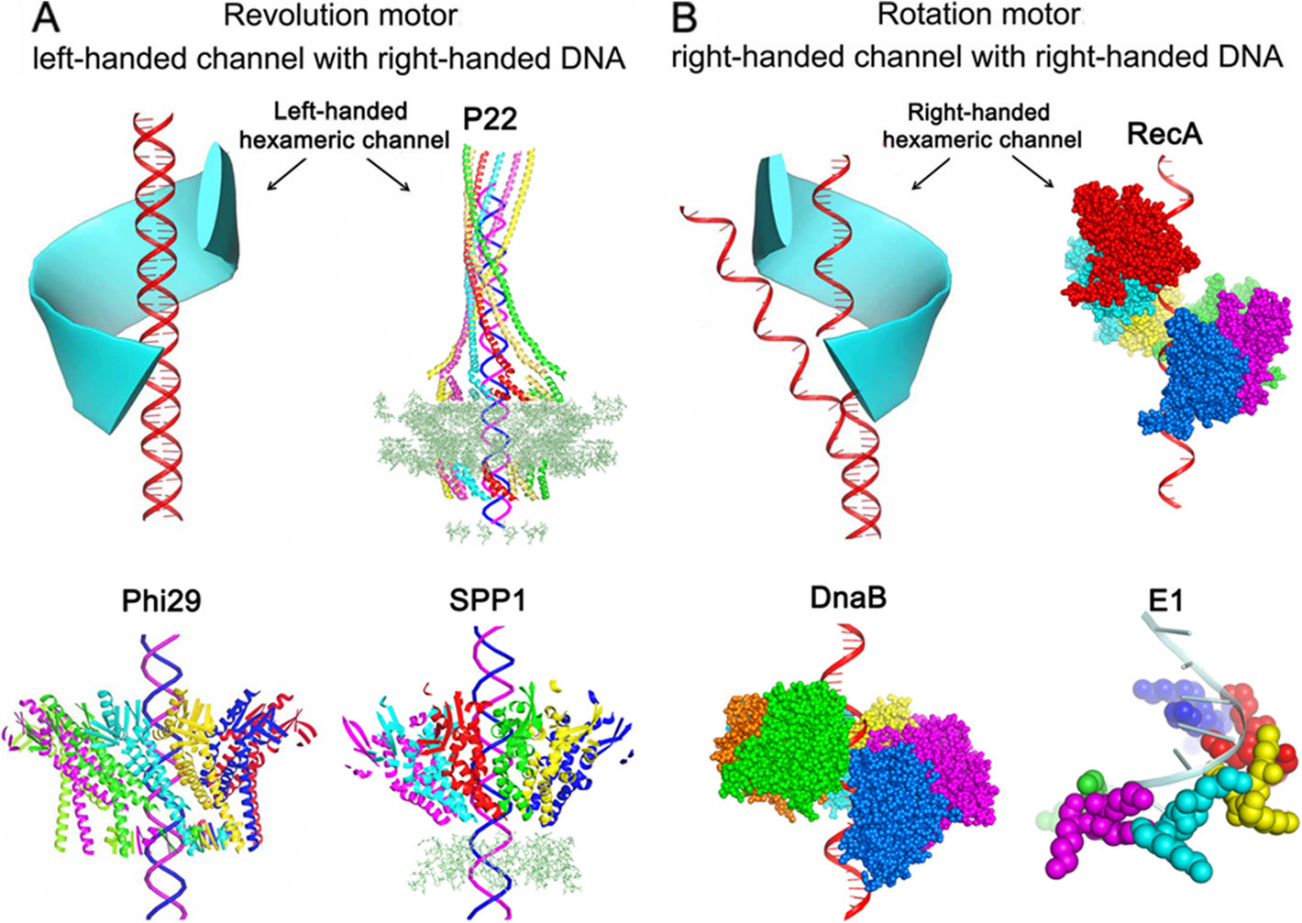
**Chirality comparison of revolution and rotation motors. (A)** In revolution motors, the right-handed DNA revolves within a left-handed channel, such as in the connector channels of bacteriophage Phi29 [46], P22 [45], and SPP1 [65]. **(B)** In rotation motors, the right-handed DNA rotates through a right-handed channel *via* the parallel thread, with RecA [76], DnaB [69] and E1 helicase [79] shown as examples. For E1 helicase, only the inside right-handed hairpin staircases that traces along the ssDNA are shown.

From mechanistic and physical standpoints, revolution motors depend upon a left-handed channel while rotation motors require a right-handed channel, to match the right-handed orientation seen in both B-type DNA and A-type DNA helices. Recently, it has been reported that the anti-chiral arrangement between the Phi29 channel and the dsDNA helices facilitates the revolution of the dsDNA for uni-directional translocation during packaging [50,54]. Analysis of the crystal structures of the motor channel of SPP1 [65], T7 [75], HK97, P22 [45], and Phi29 [46] revealed that all of these motor channels display the anti-chiral arrangement between the channel and the DNA helices. The helical domains of the 12 protein subunits aligned to form the connector channels in all of these phages are tilted at 30° left-handed relative to the vertical axis of the channel, resulting in a configuration that runs anti-chiral to the right-handed dsDNA helices during packaging (Figures 4 and 5A). This structural arrangement greatly facilitates the controlled motion, supporting the conclusion that dsDNA revolves, instead of rotating, through the connector channel without producing coiling or torsional forces while touching each of the 12 connector subunits in 12 discrete steps of 30° transitions for each contact [50].

Sequence alignments do not show apparent homology among the portal proteins of SPP1, T7, and HK97 family phages. Protein sizes also vary among different bacteriophages, ranging from 36 kDa (Phi29 gp10), 57 kDa (SPP1 gp6), 59 kDa (T7 gp8), to 94 kDa (P22 gp1) [44,75]. However, these portal proteins assemble into a propeller-like structure composed of 12 subunits with a central channel that acts as a valve for DNA translocation, and they all share very similar three-dimensional structures with several conserved regions that serve a common function in DNA packaging. Secondary structure prediction was carried out in search of structural similarities. The predicted secondary structures matched almost perfectly with the known 3D arrangements, confirming the validity of the results. Among almost all of the portal proteins, a very similar pattern of strands and helices with comparable spacing and length (Figure 4A) was found, particularly a sequence of α-β-α-β-β-α stretch. Detailed analysis of quaternary structures has revealed that the 30° tilted helix exists in all portal proteins of P22, SPP1, Phi29, T7, and HK97 family phages (Figure 4B). Further mapping studies have revealed that the position of the 30° tilt in the quaternary structure is located at the same conserved sequence at the last alpha helix of the α-β-α-β-β-α stretch (Figure 4A), indicating that this 30° anti-chiral arrangement plays a critical role in dsDNA packaging as it has been conserved by evolution.

As aforementioned, the rotation motors should have a right-handed channel to ensure parallel threading to the right-handed DNA. Indeed, crystal structure studies of helicase-DNA complexes have verified the right-handed spiral configuration of the hexameric protein-DNA complex (Figure 5) [69,76,77]. In some cases, the motor channels adopt right-handed chirality only when the ring is distorted while in complex with DNA, such as RecA filament [76] and DnaB, which functions in a nonplanar hexameric conformation during their movement relative to DNA [69], otherwise, it remains as a closed symmetrical ring as observed in the absence of DNA [78]. E1 helicase also adopts a right handed staircase pattern in the conformation of side chains when bound with DNA [79]. All of these crystallographic studies suggest that these right-handed complexes use the rotation mechanism (or a mechanism similar to a rotation mechanism for RecA, where its monomers assemble on one end of the filament and disassemble on the other). It is also possible that the gp16 ATPase in the Phi29 dsDNA packaging motor also adopts a nonplanar filament assembled from continuously spiral hexamers (or assembled from dimers) rather than a planar closed ring during the DNA packaging, however, gp16 ring might display a left-handed configuration (Figure 5A).

### Common force generation mechanism of dsDNA translocation motors in bacteria, eukaryotic and prokaryotic viruses

The recently discovered revolution motors use a hexameric ATPase to drive the advance of dsDNA in a sequential manner. Cellular dsDNA translocases also assemble into hexameric structures [4,5,80]. The cellular components that show the strongest similarity to phage revolution motors are found in the bacteria FtsK and SpoIIIE family of the ASCE DNA motor group [56-58,63]. Available evidences [58,60] lead to our hypothesis that FtsK and SpoIIIE motors also use a revolution mechanism to translocate dsDNA without rotation. Indeed, translocation of dsDNA by FtsK at a rate of 1.6-1.75 base per ATP [58,60] quantitatively agrees with the Phi29 DNA packaging motor in which each ATPase subunit uses one ATP to package 1.75 nucleotide [9,34,50,53,54]. Sequence studies of motor components of large eukaryotic dsDNA viruses, such as *Acanthamoeba poylphaga mimivirus* (APMV), and vaccinia viruses contain a dsDNA translocation motor that is similar to that of the FtsK-HerA superfamily [63,81,82], suggesting that these viruses also use the revolution mechanism for dsDNA packaging. Computation studies provide strong evidence that Phi29 DNA packaging motor ATPase gp16, FtsK, and the mimivirus motor ATPase all fall into the FtsK-HerA superfamily with a configuration of a hexameric motor ring [63,81].

As shown in this report, quaternary structure analysis revealed that a left-handed, 30° tilted helix arrangement exists in the channel wall of dsDNA bacteriophages P22, SPP1, Phi29, T7, and HK97. During revolution of dsDNA through the channel, it advances by touching the side of the channel wall instead of proceeding through the center of the channel [50,83]. As a result, the 30° left-handed direction for each transition between two connector subunits and the 30° alteration for dsDNA to advance 1/12 of helical pitch neutrally, resulting in a zero gain, that is, no rotation occurs for the dsDNA during the translocation. The proposed model of 60° per step of the FtsK hexamer (360° ÷ 6 = 60°) [58] agrees with the finding of 30° per step within the dodecamer connector channel (360° ÷ 12 = 30°) of all dsDNA bacteriophages and 60° per steps within the Phi29 hexameric ATPase gp16 [9,50,53,54].

Channel size and chirality are key factors in the identification of translocation motor types which can reveal the motor mechanism. The channel size is a physical confinement that can be used to distinguish revolution motors from rotation motors. As shown in this report, examination of the motor structures from X-ray crystallography reveals that revolution motor channels are larger than rotation motor channels. The finding that heteroduplex loop structures up to 19 bases can translocate through the phage lambda portal with the same efficiency as genome packaging [84] is another indication that the channel of lambda is wider than the dsDNA as well.

Revolution motors make contact with only one strand of the dsDNA in the 5′ to 3′ direction in order to revolve along the connector channel, which has been evidenced in various motors such as Phi29 [85,86] and T4 [87]. The model that dsDNA interacts with the internal surface of the hexameric ring [50,54] is in agreement with the observation in FtsK that only one strand of the dsDNA touches the internal wall of the motor channel [57,58].

Besides, further analysis of the crystal structures of phage connectors among SPP1, P22, and Phi29 [46,54,88,89] revealed four potential-relaying electropositive lysine residues lying on the predominantly negatively charged connector channel surface. Although these four positively charged layers are nonessential for motor DNA packaging activity [89], they are reported to influence DNA translocation [89,90]. Investigations into the detailed interaction of lysine residues with the bacteriophage genome during translocation revealed that the force generation mechanism of the relaying layers inside the channel wall altered the speed of DNA translocation resulting in four pauses [9,54]. The interaction between these positively charge lysine rings and the negatively charged phosphate backbone of the DNA suggests that SPP1, P22 and Phi29 viral dsDNA packaging motors involve an electrostatic force in DNA translocation.

Furthermore, it has been reported that the dsDNA spooling in the filled capsid is a common phenomenon in all the T7, Phi29, ϵ15, P22, and λ phages [91-94]. The revolution mechanism explains this spooling phenomenon. During packaging of DNA [50,54], dsDNA will spool within the procapsid naturally as a result of the revolution process. Since rotation is not involved, no coiling is generated and no free DNA terminus is required during spooling. Initially, extra room results in a random arrangement of the entering DNA, however, towards completion of packaging it spools tighter and tighter due to revolution, which results in a more ordered orientation of the dsDNA [91-94]. In addition, the reported revolution mechanism of phage DNA packaging motors is also consistent with recent cryo-EM imaging studies showing that the T7 dsDNA core tilts from its central axis [83].

### DNA twists rather than rotates due to motor channel conformational changes during DNA translocation

**Figure 6:**
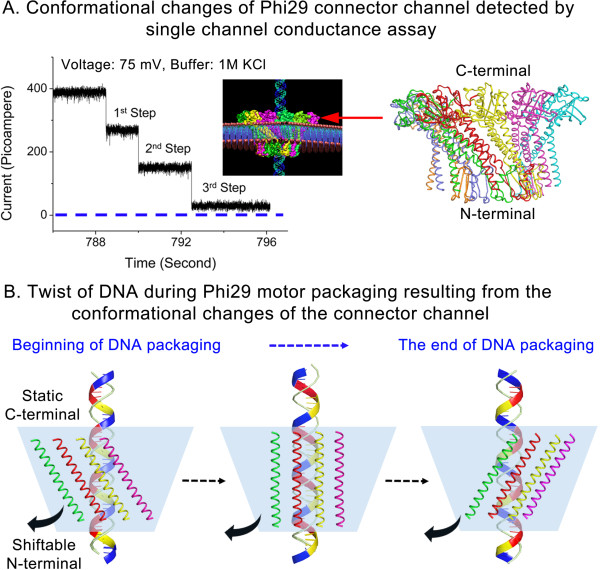
**Illustration of the mechanism of dsDNA twisting during DNA packaging due to conformational changes of the Phi29 connector channel. (A)** Discrete three step-wise conformational changes of Phi29 connector channel were detected by single channel conductance assay with the connector embedded in lipid bilayer. The external view of the crystal structure of the connector channel is shown on the right. **(B)** The C-terminal of the connector inside the procapsid is more static than the external N-terminal. As a result, the N-terminal of the connector may shift leftwards during the DNA packaging, leading to the clockwise twist of the DNA that aligns within the connector channel wall.

Many connector channels of dsDNA bacteriophages (Figure 4) adopt a left-handed channel wall to facilitate one-way traffic during dsDNA packaging into pre-assembled protein shells [52,54]. The conformational changes of the channel have been reported associated with this packaging process [95,96]. Such conformational changes allow conversion of the left-handed connector after completion of DNA packaging towards the opposite configuration, thus facilitating DNA one-way ejection into host cells for infection. Indeed, three steps of conformational changes of the Phi29 connector were detected (Figure 6A) [96], and discovered in the DNA packaging motor of SPP1 (Wang and Guo, unpublished data). Noticeable conformational differences between isolated Phi29 connectors and connectors in virions confirm such a structural transition after DNA packaging [95]. In the Phi29 crystal structure, the connector subunit displays a left-handed 30° tilt (Figure 4). However, when treated as a rigid body, the crystal structure clearly does not fit into the cryo-EM density maps, indicated by a correlation coefficient as low as 0.55. After manual adjustments, the correlation coefficient was improved to 0.70, resulting in a 10° twist of the connector towards the connector axis [95]. On the other hand, the N-terminal external region is difficult to adjust to fit as a rigid body into other parts of the connector density. It was found that the N-terminal external region underwent significant conformational shift in the DNA-filled capsid [95]. It was concluded that angular twisting and restructuring of the connector core subunit are promoted by the interactions among Phi29 DNA and its structural proteins [95]. Due to the dsDNA alignment with the channel wall [9,10,50,53,54] and the relatively static C-terminal internal region, a significant conformational shift in the N-terminal external region then results in a clockwise twist of the dsDNA when viewed from the C-terminus (Figure 6).

Recently, it has been reported that a small angular twist of 1.5 degree per nucleotide was observed during dsDNA packaging in Phi29 [97]. Observation of such a small angular deviation per nucleotide can be explained by these conformational changes of the connector (Figure 6). As evidenced above [95], if the N-terminal external region is shifted more significantly than the internal C-terminal region, a leftward twist of the DNA will occur during revolution along the connector channel (Figure 6B). This is in agreement with the observed clockwise twist of 1.5 degree per nucleotide relative to the C-terminus of the connector [97]. The reported twist of 1.5 degree per nucleotide or 15.75° per helical pitch of 10.5 bp [97] during dsDNA packaging cannot be taken as rotation mechanism in which 360° per pitch or ~34° per base pair are required. Furthermore, the reported increase in the frequency of DNA twisting per nucleotide with increase in capsid filling, is in agreement with the observation that the conformational change of the channel accelerates towards the end of the packaging process [96] (Figure 6A). This is logical since the dsDNA is aligned with the wall of the connector channel, and when DNA packaging is close to completion, a final conformation will be adopted and a more obvious twisting will be observed to prepare the channel for DNA ejection toward host infection.

### Single-molecule real-time imaging and force spectroscopy revealed that no rotation occurs during DNA translocation

**Figure 7:**
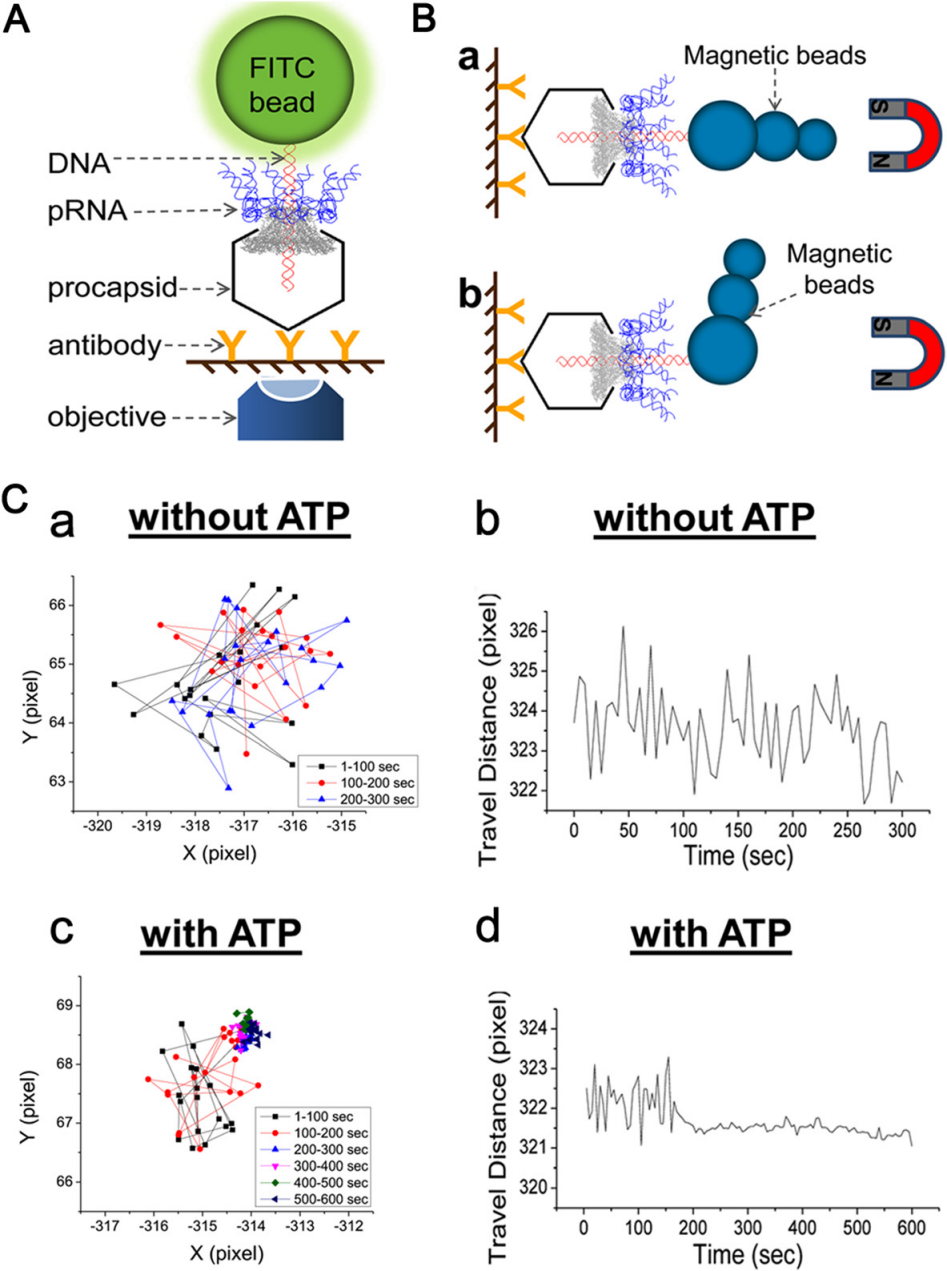
**Demonstration of no DNA rotation by real-time direct observation of single motor DNA packaging.** Procapsid was immobilized in glass and the distal end of dsDNA was tethered to a bead. DNA is packaged vertically **(A)** or horizontally **(B)** towards the slide surface (graphic is not drawn to scale). **(C)** The motion of the bead is tracked during DNA packaging without (a and b) and with (c and d) the addition of ATP to the sample. The motion of the bead ceased at later times only when ATP was added (c) and (d) due to the physical restriction of DNA being completely packaged. (a) and (c) show the trajectories of the bead. Different colors represent different time ranges during the translocation. (b) and (d) show the changes in beads travel distance versus time.

**Figure 8:**
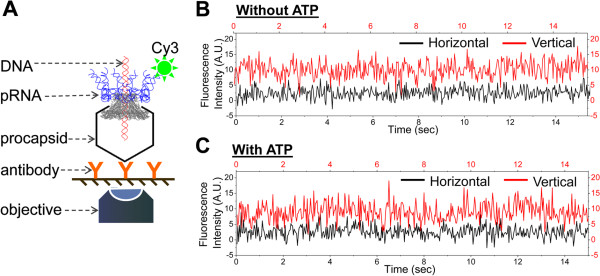
**Single molecule polarization detection to investigate motor rotation. (A)** Experiment design of single molecule polarization detection on motor pRNA rotation during DNA packaging. The motor was stalled by γ-S-ATP and the rotation of pRNA ring can be excluded since no anti-correlated signals of a single Cy3 fluorophore in horizontal (H) or vertical (V) channels were observed. **(B and C)** Typical time trajectories of Cy3 fluorescence intensity in horizontal (black) and vertical (red) channels without **(B)** and with **(C)** the addition of ATP to restart the packaging.

In order to validate the model of revolution without the need for rotation, several single-molecule imaging experiments were carried out (Figures 7 and 8). A micrometer-sized fluorescence bead was attached to the distal end of the Phi29 genomic dsDNA. DNA translocation was directly observed in real-time by single-molecule imaging microscopy to detect fluorescence images revealing the displacement of the bead [49,51]. No rotation was found in these traces (Figure 7). To exclude the possibility that the lack of rotation is a result of bond freedom between the beads and DNA or due to the difficulty in optical discrimination due to the spherical nature of the beads, a cluster of magnetic beads was attached to the end of the Phi29 DNA to generate a label with an asymmetric shape (Figure 7B) [49]. Experiments using different setups for DNA packaging in a vertical (Figure 7A) and horizontal orientation (Figure 7B) [49] have been repeated many times and no rotation of DNA was observed. Polarization studies have been used to study biomotors such as T4 helicase [72]. The polarization analysis of Phi29 DNA packaging motor did not find a rotation phenomenon either (Figure 8).

The mechanism where no DNA rotation is required during packaging is further supported by the observation that in bacteriophage T4, both DNA ends are tethered to the portal throughout DNA packaging once the packaged DNA persistence length of about 500 bp is reached, suggesting that no rotation is needed and DNA does not get tangled up [87,98]. All these observations support a revolution mechanism for phage DNA packaging without the need for rotation.

## Conclusion

The revolution mechanism is a common feature shared by many DNA translocation motors. Inspections of structural data from eukaryotic and prokaryotic dsDNA translocases suggest that revolution and rotation motors can be distinguished by measuring the size and chirality of the DNA translocation channel. The channel of revolution motors are larger than 3 nm, while the channels of rotation motors are smaller than 2 nm in diameter. Revolution motors use a left-handed channel to drive the right-handed dsDNA in an anti-chiral arrangement, while some rotation motors use parallel threads with a right-handed channel. Revolution motors hold both strands of the dsDNA within the channel, while some rotation motor hold only one strand of the DNA inside the channel [5,64,67,69-71]. Such revolution motors are void of dsDNA coiling [9,50,54,55]. A small-angle left-handed twist of dsDNA, which is aligned with the channel, takes place due to the conformational shifts of the motor channel from a left-handed configuration for DNA entry to a right-handed configuration for DNA ejection for host cell infection, however, no dsDNA rotation is required for DNA packaging.

## Materials and methods

### Incorporation of the connector channel into a planar bilayer lipid membrane

The method of inserting the connector with reconstituted liposomes into a lipid bilayer has been reported previously [99]. Briefly, a Teflon film partition (aperture 200 μm in diameter) was used to separate a bilayer lipid membrane chamber (BLM) into *cis-* and *trans*- compartments. The aperture was painted two times with 0.5 uL of 3% (w/v) DPhPC n-decane solution, and the two compartments were filled with conducting buffer (1 M NaCl or 1 M KCl, 5 mM HEPES, pH 7.4). After formation of the lipid bilayer on the aperture, the lipid/connector complexes were added to the chamber and allowed to fuse with the planar lipid bilayer.

### Construction of tetra-stranded DNA

Five strands were custom ordered from IDT, with the following sequences: Strand-1: 5′-CGC AGA CAT CCT GCC GTA GCC TGA GGC ACA CG-3′; Strand-2: 5′-CGT GTG CCT CAC CGA CCA ATG C-3′; Strand-3: 5′-GCA TTG GTC GGA CTG AAC AGG ACT ACG CTG GC-3′; Strand-4: 5′-GCC AGC GTA GTG GAT GTC TGC G-3′; Strand-5: 5′-TC AGT GGC TAC GGC ACC GT-3′. The five strands were annealed in stoichiometric ratio in TMS (Tris-magnesium saline) buffer (50 mM Tris–HCl, pH8.0, 100 mM NaCl and 10 mM MgCl_2_) and purified in 12% (w/v) native PAGE, following reported procedures [100].

### Single channel conduction assays for each membrane inserted connector channels

A pair of Ag/AgCl electrodes was connected directly into the *cis-* and *trans*- compartments to measure the current traces across the lipid bilayer membrane. The current trace was recorded using an Axopatch 200B patch clamp amplifier coupled with the Axon DigiData 1322A analog-digital converter (Axon Instruments) or the BLM workstation (Warner Instruments). All voltages reported were those of the *trans*- compartment. Data was low band-pass filtered at a frequency of 1 kHz, and acquired at a sampling frequency of 10–100 kHz. The Patch clamp 9.1 software (Axon Instruments) was used to collect the data, and the software Origin Pro 8.0 was used to analyze all the data.

### Direct observation of DNA translocation

The stalled packaging intermediates containing biotinylated DNA were prepared by using non-hydrolyzable γ-S-ATP [101]. The intermediates were then immobilized to perfusion chambers built from glass slides and coverslips (Figure 7). The 0.53 mm fluorescent streptavidin microspheres (Bangs Laboratories Inc.) were bound to the protruding, biotinylated DNA end of the intermediates. After restarting the packaging reaction by adding gp16 and ATP [101], an individual DNA-packaging event was observed. Epi-illumination was used. Sequential images with 8-bit digital resolution were recorded at 1 frame per second for 600 s. The pixel resolution of the images was 0.26 mm/pixel.

## Abbreviations

DsDNA: Double stranded DNA; ASCE: Additional strand catalytic E; EMSA: Electrophoretic mobility shift assays; smFRET: Single molecule fluorescence resonance energy transfer.

## Competing interests

Peixuan Guo is a co-founder of Kylin Therapeutics, Inc., and Biomotor and RNA Nanotech Development Co. Ltd.

## Authors’ contributions

GMDD, ZZ, LH, FH and CS carried out the experiments and participate the manuscript preparation. HZ carried out the single-molecule experiments. SW carried out the sequence alignment. OT participated in the data analysis. PG conceived the concept, designed the experiment and wrote the manuscript. All authors read and approved the final manuscript.

## Authors’ information

Gian Marco De-Donatis and Zhengyi Zhao serve as co-first author.
